# The Role of Stereotactic Body Radiotherapy in Oligometastatic Non-Small Cell Lung Cancer

**DOI:** 10.3390/curroncol31120588

**Published:** 2024-12-15

**Authors:** Benson Wan, Magali Lecavalier-Barsoum

**Affiliations:** 1 Division of Radiation Oncology, Faculty of Medicine, McGill University, Montreal, QC H4A 3J1, Canada; 2 Department of Oncology, Faculty of Medicine, McGill University, Montreal, QC H3T 1E2, Canada; 3 Department of Radiation Oncology, Jewish General Hospital, McGill University, Montreal, QC H3T 1E2, Canada

**Keywords:** SBRT, NSCLC, oligometastasis

## Abstract

Non-small cell lung cancer (NSCLC) is a major cause of mortality in Canada, with many patients presenting with metastatic disease. The oligometastatic state (OM-NSCLC) may be amenable to cure using aggressive local consolidative therapies. Stereotactic body radiotherapy (SBRT), which entails the utilization of a high dose of radiation in one or few fractions, has many benefits in this setting, including its applicability in varied patient populations to ablate lesions in varied anatomical locations. It has also been demonstrated to prolong the time to next-line systemic therapy, to reduce financial burden, to improve quality-adjusted life years, and reduce adverse events caused by these lesions. This review outlines the published phase II and III trials that have already demonstrated the utility of SBRT in OM-NSCLC, as well as the many ongoing trials aiming to further define its role, including the largest phase II/III trial to date, NRG-LU002. Overall, SBRT appears to improve outcomes when combined with a broad range of standard-of-care therapies and is generally well tolerated; however, careful patient selection is necessary to maximize benefits while minimizing harm. Ongoing trials will help define the optimal patients for SBRT and the best timing for this intervention.

## 1. Introduction

Lung cancer continues to be the leading cause of cancer-associated mortality in Canada, with most deaths being attributed to non-small cell lung cancer (NSCLC) [1]. Roughly half of all new lung cancer patients present with metastatic disease [2]. Recently, there has been significant progress towards managing NSCLC, including screening, diagnosis, and research in molecular aberrations that form the basis for targeted therapies, as well as immunotherapy, with 5-year overall survival (OS) rates in the order of 15–50% now being reported [3].

The clinical state of oligometastatic (OM) disease was first described in 1995 by Hellman and Weichselbaum, in which metastases are concentrated to a limited number of organs [4]. They proposed that this state was distinct from advanced metastatic disease; as such, these patients could possibly attain a cure from disease with metastasis-directed local therapy in combination with systemic therapy. Metastases-directed local consolidation therapies (LCTs) employed in the OM setting include radiation therapy, surgery, radiofrequency ablation, radioembolization, and chemoembolization. One form of radiotherapy employed is stereotactic body radiotherapy (SBRT), defined as an external beam radiotherapy that accurately delivers a high dose of radiation in one or few (typically up to five) treatment fractions to an extracranial target [5].

SBRT and its intracranial counterpart, stereotactic radiosurgery (SRS), have both curative and palliative applications. Compared with other LCT techniques, SBRT has some advantages that can be leveraged for certain patients. In the context of oligometastatic disease, SBRT can be used to achieve local control, particularly when other modalities are not feasible or are technically difficult. For instance, SBRT is a non-invasive treatment and can be given to patients with medical comorbidities who would be considered non-operative. Furthermore, SBRT can target lesions situated near vasculature which may act as heat sinks that would complicate embolization-based techniques. The side effect profile SBRT is also favorable as compared to other techniques, with low rates for severe toxicities.

This article aims to discuss some of the existing literature on the use of LCT, specifically SBRT, in OM-NSCLC. It will review the definition of OM disease, the rationale of using LCT, completed and ongoing trials, and a discussion on the role of SBRT in OM-NSCLC.

## 2. Methods

To explore the role of SBRT in OM-NSCLC, several completed phase II and III studies and ongoing trials were highlighted. To identify these articles, a literature search was performed using the National Institutes of Health’s National Library of Medicine using the search terms [(“SBRT” OR “SABR” OR “RADIOTHERAPY”) AND (“Oligomet*”) AND (“NSCLC”)]. Additionally, presentations and abstracts presented at the American Society of Clinical Oncology (ASCO) Annual Meeting 2024 were also chosen for discussion. Of note, as this is not a systematic review, we then used our discretion to select certain studies of interest for this article.

## 3. Oligometastatic Paradigm

Currently, OM disease is defined as a limited metastatic disease in the setting of either newly diagnosed disease or after first-line systemic therapy without evidence of progression [6]. Historical OM trials have varied definitions of OM disease, typically ranging from up to three to five metastatic deposits with limitations on the maximum number of lesions in one organ system. Newer definitions have been increasing the number of allowable metastatic deposits to ten. With advancements in the treatment arsenal against metastatic lung cancer, including traditional cytotoxic regimens, immunotherapies and tyrosine kinase inhibitors, there is also increased interest in leveraging local therapies to achieve cure in OM-NSCLC.

When describing OM disease, it is important to note that there are several terms that describe clinical states that are similar yet distinct. Oligoprogressive disease describes disease in which there is radiological evidence of progression of disease in either a limited number of existing or new sites, while on systemic therapy [7]. Although not the focus of this article, data from phase II trials demonstrate that targeting the sites of oligoprogression in metastatic patients, including those with metastatic NSCLC, may lead to improvements in progression-free survival (PFS) and local control [8,9]. Oligopersistent disease is another term referring to the persistence of a limited number of lesions in patients receiving active systemic treatment, regardless of the initial number of lesions. Oligorecurrence describes development of a limited number of metastases after initial radical treatment for non-metastatic disease. Finally, the polymetastatic state describes metastatic disease with six or more metastases [10].

The most commonly reported benefits of metastasis-directed SBRT are progression-free survival (PFS) and overall survival (OS). However, the use of SBRT in select OM patients may be beneficial in other manners as well. For instance, local therapy could lead to improved local control, which may delay the switch to next-line therapy or delay the need for additional salvage systemic therapies [11]. This has many implications on both the health care system in reducing financial toxicity and on the patient through means such as quality of life. Financial analyses performed on previously completed trials, such as the SABR-COMET trial, have demonstrated that SBRT is cost-effective and provides improvements in quality-adjusted life years when added to the standard of care treatment [12,13]. Additionally, oligometastases that progress may become symptomatic or lead to adverse events such as pain crises, superior vena cava syndrome, cord compression, and bleeding. Treating these lesions prior to the onset of symptoms may provide benefits, as recently demonstrated in a randomized clinical trial on patients with high-risk asymptomatic bone metastases [14].

## 4. Current Data on SBRT in OM-NSCLC

Table 1 summarizes some randomized phase II and phase III clinical trials comparing the standard of care with the addition of local consolidative therapy [15,16,17,18]. The SABR-COMET trial was not specific to OM-NSCLC and included patients with other pathologies, whereas the other studies were specific to OM-NSCLC. In the Gomez trial, the majority of patients received SBRT/hypofractionated RT as an LCT; however, some patients underwent surgery. All trials demonstrated an improvement in PFS and OS with the addition of an LCT. Furthermore, Iyengar et al. [18] demonstrated the absence of in-field recurrences and a reduced number of recurrences overall, albeit in a very limited sample size. Across all four trials, there was one SBRT-associated grade 5 toxicity that occurred in the SABR-COMET study; treatment was otherwise well tolerated with low rates of grade 3 or higher adverse events.

Table 2 summarizes a select few other studies in terms of the role of SBRT in OM-NSCLC [19,20,21,22]. Of note, the NRG-LU002 was recently presented at the American Society of Clinical Oncology Annual Meeting in 2024, which is the largest randomized phase II/III clinical trial to date on LCT in OM-NSCLC, with 215 patients enrolled. Of note, over 90% of patients in this trial received immunotherapy-based systemic therapy, which is distinct compared to previous studies. The 1- and 2-year PFS and OS rates were not statistically different between the maintenance plus LCT and maintenance arms. There were also more adverse events related to treatment, in particular, grade 3 pneumonitis (10% vs. 1%) and grade 2 toxicities (84% vs. 73%) in the LCT arm. Subgroup analyses and the final publication are pending. The other studies described, which were abstracts, phase I trials, or retrospective in nature, provide data that support the use of LCT in select patient populations in the context of cytotoxic therapies, targeted therapies, and immunotherapies.

## 5. Ongoing Clinical Trials of SBRT in OM-NSCLC

Numerous trials that aim to investigate the role of SBRT and OM-NSCLC in specific clinical situations are pending.

SARON is a randomized controlled phase III trial on OM-NSCLC without driver mutations, in which patients may have up to three sites of metastatic disease, at least one of which must be extracranial [23]. Participants are randomized to either standard platinum-doublet chemotherapy alone or chemotherapy with radical radiotherapy to the primary tumor and SBRT to all sites of metastatic disease.

The NORTHSTAR trial is a randomized phase II trial that enrolled EGFR-mutated NSCLC patients with either stage IIIB or stage IV disease, randomized to Osimertinib alone versus Osimertinib induction followed by LCT, consisting of surgery and/or radiation therapy [24]. One of this study’s secondary objectives is to analyze whether osimertinib with local consolidative therapy improves PFS compared to osimertinib alone in an OM subset of patients with one to three metastases.

The LONESTAR trial is a randomized, phase III trial enrolling patients with metastatic NSCLC; the study arms are nivolumab and iplimumab, with or without local consolidative therapy [25]. One of its primary objectives is to determine whether LCT prolongs overall survival in the subgroup with oligometastatic disease with up to three metastases.

The ImmunoSABR trial is another randomized, phase II trial comparing the immunocytokine L19-IL2 alone with the immunocytokine with SBRT to all sites of disease in metastatic NSCLC in the first-, second-, or third-line setting [26]. Both OM and polymetastatic patients will be included; however, a maximum of five lesions may be treated with SBRT in all patients. Their primary endpoint will be PFS at 1.5 years, and their secondary endpoints include OS, toxicity, QoL, and the presence/absence of abscopal effect.

The SABR-COMET-3 and SABR-COMET-10 trials are phase III trials enrolling patients with oligometastatic cancers with between one and three metastatic lesions and between four and ten metastatic lesions, respectively [27,28]. Patients enrolled in either trial will be randomized to receive the standard of care treatment with or without SBRT to all sites of known disease. These studies will include some NSCLC patients who may be on systemic immunotherapy or targeted therapies; however, this will not be the only patient population of interest. Both trials note OS as their primary endpoint.

## 6. Discussion

Several published phase II and III clinical trials have shown that LCTsw, including SBRT, may improve outcomes when utilized in conjunction with standard-of-care therapy. This has been demonstrated in the published trials performed by Wang et al. [15], Gomez et al. [16], and Iyengar et al. [18], with improvements being seen in both PFS and OS. In these studies, up to 5 oligometastatic lesions were treated with a variety of doses and fractionations. Metastatic deposits in a variety of organs were treated in these studies, in addition to the lung primary and regional nodes, suggesting that these treatments can be used broadly in OM-NSCLC patients. The patients studied also tolerated treatment well, with one instance of grade 5 toxicity being attributable to SBRT in the SABR-COMET study and other SBRT-related toxicities of grade 3 or less occurring in one third of the patients treated or fewer.

Current studies have primarily included patients on cytotoxic regimens and targeted therapies; however, there are few published data investigating the addition of LCT on immunotherapy-based regimens. With the data from the KEYNOTE-024 and KEYNOTE-042 studies demonstrating the benefit of immunotherapy over standard-of-care chemotherapy with patients with PD-L1 expression, first-line immune checkpoint inhibitor therapy is becoming more common for patients without driver mutations [29,30,31]. Newer studies, such as the NRG-LU002, LONESTAR, and ImmunoSABR are aiming to capture the evolving landscape of systemic therapies.

While we await the final published data from the NRG-LU002 trial, the negative data published at ASCO 2024 were striking for several reasons. To date, this is the largest phase II/III clinical trial on OM-NSCLC, and its inclusion of patients on immune checkpoint inhibitor therapy reflects current treatment practices. The subgroup analysis may shed light on the discrepancy of outcomes from this study compared to previous studies. However, some theories on the lack of LCT-associated benefit in this cohort include the increased efficacy of immunotherapy which reduces the benefit of LCT, the unique biology of residual disease after upfront immunotherapy, and possible patient differences in the study arms. Although only preliminary data have been presented, these results suggest that LCT should not routinely be offered to all patients; rather, an informed, multidisciplinary decision should be made on an individualized basis. The risk of the considered treatment should be weighed against the risk of symptoms or the development of adverse events in case of progression of the lesion(s).

Another key point is that different patient populations may demonstrate benefits from LCT to varying degrees. For instance, a patient that is on very effective systemic therapy may not benefit much from LCT, as continued systemic therapy may provide disease control itself. However, a patient that is responding poorly to their systemic treatment may benefit more from an LCT like SBRT. With the advancements in targeted therapies and immunotherapies, survival in terms of OM-NSCLC can potentially be greatly improved. As such, there is a need for additional trials that evaluate the impact of SBRT on OM-NSCLC patients undergoing newer regimens, as data from older trials may not necessarily be confidently extrapolated.

The best timing of when to employ LCT in relation to systemic therapy remains to be defined. SBRT and other LCT techniques are only able to treat macroscopic, clinically detectable diseases. In the literature reviewed here, SBRT has been given at various time points ranging from upfront prior to systemic treatment to several weeks after the completion of first-line treatment. Some early data support that LCT can be given concurrently with immunotherapy without additional toxicity as compared to being given sequentially [22]. Given this heterogeneity, there has yet to be an optimal time window for LCT. With delayed LCT, it would also be possible to identify patients that demonstrate progression in multiple sites rather than in a limited number of sites and who, thus, are not truly oligometastatic. As such, there may be unique benefits associated with both immediate and delayed LCT.

Published phase II-III trials show clear role for LCT techniques such as SBRT in attaining local control in OM-NSCLC patients. This has been demonstrated to be beneficial in improving survival in patients, cost-effective, and generally well tolerated. However, with the rapid advancements and changes in systemic therapies that have drastically improved patient survival, the role of LCT needs to be redefined. It is unlikely that LCT will be removed from oncologists’ arsenal of treatment options; however, treating all lesions indiscriminately may not be judicious. Once data from newer randomized OM-NSCLC trials investigating the use of LCT are published, this will allow the multidisciplinary oncology team to better select which patients and which lesions should be treated.

## Figures and Tables

**Table 1:** Selected phase II and phase III trials highlighting the potential role of SBRT and LCT in OM-NSCLC.

Study	Sample Size	Inclusion/Exclusion Criterion	Dose and fx	Results	Treatment-Related Toxicities
SINDAS Wang et al., 2023 [15] Randomized, Phase 3 Trial	A total 133 patients, 68 RT, 65 no RT	EGFR-mutated NSCLC Synchronous oligometastatic (new dx, tx naïve, ≤5 lesions, ≤2 in one organ system) No brain mets Involved regional nodes were not counted as mets	Upfront RT, 25–40 Gy in 5 fx, prior to TKI, maximum 3 week dose-interruption Primary tumor and regional nodes also treated with 5 fractions	PFS—12.5 months vs. 20.2 months OS—17.4 months vs. 25.5 months	RT: 6% grade 3–4 pneumonitis, 15% grade 3–4 skin rash, 7% grade 3 prurutis, 2% grade 4 transaminitis, 4% grade 3–4 esophagitis No RT: 3% grade 3 pneumonitis, 14% grade 3–4 skin rash, 12% grade 3–4 pruritus, 2% grade 4 fatigue, 2% grade 4 transaminitis, 3% grade 2 esophagitis, 3% grade 3–4 pericarditis, 3% grade 4 pleural effusion
Gomez 2019 [16] Randomized, phase 2	A total of 49 patients, 25 LCT, 24 no LCT A total of 12 hypoRT, 6 surgery + RT, 2 RT and chemotherapy, 3 hypoRT and chemo, 1 surgery to all sites	Metastatic NSCLC, ≤3 disease sites after systemic therapy (≥4 cycles of platinum doublet, or ≥3 months of EGFR or ALK inhibitors) Involved thoracic nodes counted collectively as lesion Brain mets treated before randomization allowed	HypoRT: 15–20 Gy in 1 fx, 30–40 Gy in 10 fx, 50 Gy in 4 fx, 45 Gy in 15 fx, 60 Gy in 8 fx Lung and Lymph Nodes received curative doses when possible: 45–66 Gy in 4–33 fx	PFS—14.2 months vs. 4.4 months OS—41.2 months vs. 17.0 months Survival after progression—37.6 months vs. 9.4 months	LCT arm: 8% grade 3 esophagitis 4% grade 3 anemia, 4% grade 3 pneumothorax Maintenance arm: 4% grade 3 fatigue, 4% grade 3 anemia
SABR-COMET Palma et al., 2020 [17] Randomized, phase 2	A total of 99 patients, 66 SBRT, 33 no SBRT A total of 18 lung patients, 12 of whom received SBRT	Metastatic cancer, controlled primary, 1–5 metastatic lesions, maximum of 3 in one organ	30–60 Gy in 3–8 fractions to all sites of metastatic disease Single fraction 16–24 Gy allowed for brain and vertebrae	5-year OS—42.3% vs. 17.7% 5-year PFS: 17.3% vs. NR No QOL differences	SBRT arm: 5% grade 5 event (2% grade 5 pneumonitis), 2% grade 3 dyspnea, 5% grade 3 pain No SBRT arm: 3% grade 3 fatigue
Iyengar 2018 [18] Randomized, phase 2 study	A total of 29 patients, 14 SBRT, 15 no SBRT	NSCLC (primary plus up to 5 metastatic sites, no more than 3 sites in liver or lung) Partial response or stable disease post induction, RT given 21–42 days within first-line	21–27 Gy in 1 fx 26.3–33 Gy in 3 fx 30–37.5 Gy in 5 fx Primary: 45 Gy in 15 fx	PFS: 9.7 months vs. 3.5 months	SBRT: no grade 3 or higher attributable to SBRT 14% grade 1 respiratory No SBRT: 13% grade 3–4 hematologic, 7% grade 3 infectious

Abbreviations: RT—radiotherapy, EGFR—epidermal growth factor receptor, NSCLC—non-small cell lung cancer, Gy—Gray, fx—fractions, TKI—tyrosine kinase inhibitor, PFS—progression-free survival, OS—overall survival, LCT—local consolidative therapy, hypoRT—hypofractionated radiotherapy, ALK—anaplastic lymphoma kinase, SBRT—stereotactic body radiotherapy, NR—not reached.

**Table 2:** Highlighted trials with preliminary data and other trials discussing the role of SBRT/LCT in OM-NSCLC.

Study	Sample Size	Inclusion/Exclusion Criterion	Dose and fx	Results	Pertinent Treatment-Related Toxicities (Pneumonitis, Grade ≥ 3 Toxicities)
NRG LU002 Iyengar et al., 2024 [19] Randomized, phase II/III	A total of 215 patients; 134 LCT, 81 in no LCT	Metastatic NSCLC, 3 or fewer extracranial sites upon restaging after 4 cycles of first-line therapy	A total of 24 Gy in 1 fx, 30 Gy in 3 fx, 34 Gy in 5 fx A total of 45 Gy in 15 fx to primary	1-year PFS: 51.5% vs. 48% 2-year PFS: 40.1% vs. 35.9% 1 year OS: 76.5% vs. 75.8% 2 year OS: 54.1% vs. 58.1%	LCT: 84% with grade ≥ 2 events, 10% grade 3 pneumonitis, 15% grade 4 events, 8% grade 5 events No LCT: 73% with grade ≥ 2, 1% grade 3 pneumonitis, 15% grade 5 events, 6% grade 5 events
NCT03275597 Bassetti et al., 2021 [20] Phase 1b study—Abstract	A total of 17 patients; 15 non-squamous	Metastatic NSCLC, 1–6 extracranial metastatic sites, no actionable driver mutation, no prior immunotherapy	A total of 30–50 Gy in 5 fx to all sites of disease, followed by Durvalumab + Tremelimumab	OS and PFS not reached	12% grade ≥ 3 hepatitis or pancreatitis, 29% grade 3 event, 6% grade 4 event
Rashdan et al., 2024 [21] Single-arm, phase II, non-randomized—Abstract	A total of 43 patients; 29 received SBRT	NSCLC, EGFR mutant, no prior treatment, no limit on number of mets, SBRT delivered to persisting lesions after 8 weeks of Osimertinib	Not reported	PFS 32.6 months OS 45.7 months Mean duration osimertinib 31.5 months	2% Grade ≥ 3 pneumonitis 2% Grade ≥ 3 pain, 2% Grade ≥ 3 paronychia, transaminitis, fatigue, hyponatremia and diarrhea
Bestvina et al., 2020 [22] Randomized, phase I	A total of 37 patients; 18 concurrent SBRT with nivolumab and ipilimumab, 19 with sequential SBRT then immunotherapy	Metastatic NSCLC, treatment naïve, no limit to number of metastases	A total of 30 Gy in 3 fx, 45 Gy in 3 fx, or 50 Gy in 5 fx	Median PFS 5.8 months Median OS not reached	Concurrent: 3% grade 5 pulmonary hemorrhage, 5% grade 3 pneumonitis, 3% grade 3 esophageal stenosis and esophagitis Sequential: 8% grade 3 pneumonitis

Abbreviations: LCT—local consolidative therapy, NSCLC—non-small cell lung cancer, Gy—gray, fx—fractions, PFS—progression-free survival, OS—overall survival, SBRT—stereotactic body radiotherapy.
